# Key factors influencing earthquake-induced liquefaction and their direct and mediation effects

**DOI:** 10.1371/journal.pone.0246387

**Published:** 2021-02-17

**Authors:** Jilei Hu, Yunzhi Tan, Wenjun Zou

**Affiliations:** 1 College of Civil Engineering and Architecture, China Three Gorges University, Yichang, Hubei, China; 2 Medical College, China Three Gorges University, Yichang, Hubei, China; China University of Mining and Technology, CHINA

## Abstract

Many factors impact earthquake-induced liquefaction, and there are complex interactions between them. Therefore, rationally identifying the key factors and clarifying their direct and indirect effects on liquefaction help to reduce the complexity of the predictive model and improve its predictive performance. This information can also help researchers understand the liquefaction phenomenon more clearly. In this paper, based on a shear wave velocity (V_s_) database, 12 key factors are quantitatively identified using a correlation analysis and the maximum information coefficient (MIC) method. Subsequently, the regression method combined with the MIC method is used to construct a multiple causal path model without any assumptions based on the key factors for clarifying their direct and mediation effects on liquefaction. The results show that earthquake parameters produce more important influences on the occurrence of liquefaction than soil properties and site conditions, whereas deposit type, soil type, and deposit age produce relatively small impacts on liquefaction. In the multiple causal path model, the influence path of each factor on liquefaction becomes very clear. Among the key factors, in addition to the duration of the earthquake and V_s_, other factors possess multiple mediation paths that affect liquefaction; the thickness of the critical layer and thickness of the unsaturated zone between the groundwater table and capping layer are two indirect-only mediators, and the fines content and thickness of the impermeable capping layer induce suppressive effects on liquefaction. In addition, the constructed causal model can provide a logistic regression model and a structure of the Bayesian network for predicting liquefaction. Five-fold cross-validation is used to compare and verify their predictive performances.

## Introduction

The selection of key factors is a critical step in any development of any model [1]. Considering too few factors will cause the model to underfit, and considering too many factors in the model will lead to overfitting. Moreover, factors with little or no effects that are added to the model will largely increase the uncertainty and complexity of the model and make it more difficult both to fit and interpret [1]. Many factors impact earthquake-induced liquefaction, mainly including seismic parameters, soil properties, and site conditions (as shown in Table 1). The contribution of each factor in these three categories to the occurrence of liquefaction is different, and the mutual influence between the factors is complicated. Therefore, identifying the key factors and screening their direct and mediation effects on the occurrence of liquefaction can largely reduce the complexity of the model and more clearly explain the influence path and mechanism of each factor, which is conducive to improving the predictive performance of the model. Table 1 summarizes almost all factors related to earthquake-induced liquefaction and their influence rules. It should be noted that many factors do not solely affect liquefaction potential (LP). For example, for the same site, the greater the moment magnitude (*M*_*w*_), the more likely the site is to liquefy, and the greater the peak ground acceleration (*PGA*) and duration (*t*); the *M*_*w*_ can indirectly promote LP through *PGA* and *t*. For the silty sand, the greater the fines content (*FC*), the more the average practical size (*D*_*50*_) decreases, and the permeability coefficient (*k*) is reduced accordingly; the increase in the *FC* and the decrease in *k* are not conducive to liquefaction, while the decrease in *D*_*50*_ is conducive to liquefaction, forming a competitive effect. However, these are only qualitative cognitions, and it is impossible to quantitatively analyse the contribution of each factor.

**Table 1:** Factors and their influence rules for earthquake-induced liquefaction.

Category	Factors	Index	Influence rule	Reference
Seismic parameter	Moment magnitude	*M*_*w*_	The bigger the *M*_*w*_, the bigger the *PGA* and *t*, the more likely to liquefy; no liquefied cases with *M*_*w*_ < 5	[2]
	Epicentral distance	*R*	The father the *R* of the site, the smaller the *PGA* and *t*, the less likely is to liquefy	
	Duration	*t*	The longer the loading lasts, the more likely the site is to liquefy	
	Predominant frequency	*f*	It plays an insignificant influence on liquefaction	
	Direction	-	It plays an insignificant influence on liquefaction	
	Amplitude	*PGA* or *PGV*	The bigger the amplitude of the site, the less likely the site is to liquefy	[3]
	Intensity	*I*	The bigger the *I*, the less likely the site is to liquefy	
Soil property	Fine or clay content	*FC*, *CC*	The non-linear relationship between liquefaction resistance and *FC* or *CC* is a concave upward parabola; *FC* or *CC* has a positive effect on LP when it less than the critical value, vice versa	[2–3]
	Soil type	*ST*	The cohesive soil and gravelly soil are usually not easy to liquefy	
	Particle size characteristic	*D*_*50*_, *C*_*c*_, *C*_*u*_	The larger the *D*_*50*_ and the better the gradation, the bigger the *k*, the less likely the soil is to liquefy	
	Relative density	*D*_*r*_ *or e*	The increase of relative density increases the liquefaction resistance	
	Over-consolidation ratio	*OCR*	The larger the *OCR*, the better the liquefaction resistance of the soil	
	Degree of saturation	*S*_*r*_	Usually, the saturated soil can liquefy	
	Plasticity index	*I*_*p*_	Liquefaction resistance decreases as the *I*_*p*_ increases	
	Soil structure	-	Well-structured soil is not easy to liquefy	
	Particle shape	-	The coarser the particles, the harder the soil is to liquefy	
	Permeability coefficient	*k*	The greater the *k*, the less likely the site is to liquefy	[4]
Site condition	Vertical stress	$\sigma_{V},\sigma_{V}^{'}$	The increase of *σ*_*V*_ or $\sigma_{V}^{'}$ increases the liquefaction resistance of the soil	[2–3]
	Groundwater table	*D*_*w*_	The deeper the *D*_*w*_, the less likely the site is to liquefy	
	Depth of critical soil	*D*_*s*_	The deeper the critical layer, the less likely the site is to liquefy	
	Thickness of the critical layer	*T*_*s*_	The occurrence of liquefaction needs a certain thickness of the *T*_*s*_, whereas simultaneously the *D*_*s*_ increases as the *T*_*s*_ increases that inhibit liquefaction	
	Deposit type	*DT*	Soil liquefaction is easy to occur near alluvial and marine plains, rivers, lakes, marshes, and depressions	
	Deposit age	*A*	The tendency of the soil to liquefy decreases over time	
	stratigraphic texture	-	It plays an insignificant influence on liquefaction resistance	
	Stress history	-	Stress history increases liquefaction resistance of the soil	
	Thickness of the impermeable capping layer	*H*_*n*_	The bigger the *H*_*n*_, the bigger the *σ*_*V*_, the less likely the site is to liquefy, whereas the occurrence of gravelly soil liquefaction requires a certain *H*_*n*_	[5]
	Drainage channel	*D*_*n*_	The site with a good drainage channel is not easy to liquefy	
	Drainage boundary	-	The better the drainage boundary, the less likely the site is to liquefy	[4]

Although there are many studies on the influence rules of various factors on liquefaction, few studies have focused on the screening of significant factors. Seed and Idriss [6] suggested five factors, namely, soil type (*ST*), relative density or void ratio, initial confining pressure, and the intensity and duration of ground shaking, for predicting soil liquefaction. Zhu [7] selected eight significant factors from 15 total factors, namely, the groundwater table (*D*_*w*_), depth of the critical layer (*D*_*s*_), normalized standard penetration blow count (*SPTN*), thickness of the impermeable capping layer (*H*_*n*_), thickness of the critical layer (*T*_*s*_), *D*_*50*_, nonuniform coefficient (*C*_*u*_) and frequency of the maximum particle size, for predicting liquefaction using the Bayesian regression method. Dalvi et al. [8] found eight significant factors, the *M*_*w*_, *PGA*, peak ground velocity (*PGV*), frequency (*f*), normalized *SPTN*, vertical effective stress ($\sigma_{V}^{'}$), dynamic shear modulus and relative density (*D*_*r*_), among 16 total factors using the analytic hierarchy process and entropy analysis method. Tang et al. [2] identified 12 significant factors from 22 total factors using the bibliometric method, and these significant factors contain almost all the important factors suggested by the above studies. Lee and Hsiung [9] presented an approach for quantifying the sensitivities of the key factors in a multilayer perceptron neural network and revealed that the *PGA* is the most sensitive factor, and the earthquake parameters (e.g., *M*_*w*_, *PGA*, etc.) are more sensitive to liquefaction potential than soil properties (e.g., *SPTN*, *FC*). However, the conclusions of these studies were different, and some research methods, such as the analytic hierarchy process and bibliometric method, were more subjective, so that the screening results were easily affected by experience or sampling, while those objective methods, such as regression methods and artificial neural networks, only considered the direct causality between the factors and liquefaction potential, whereas the mutual influence between the factors was ignored, and the mediation effects of the factors on liquefaction were not considered. Thus, the calculation of the contribution of the factors to the occurrence of liquefaction was inaccurate, which affected the identification results of the key factors.

**Fig 1:**
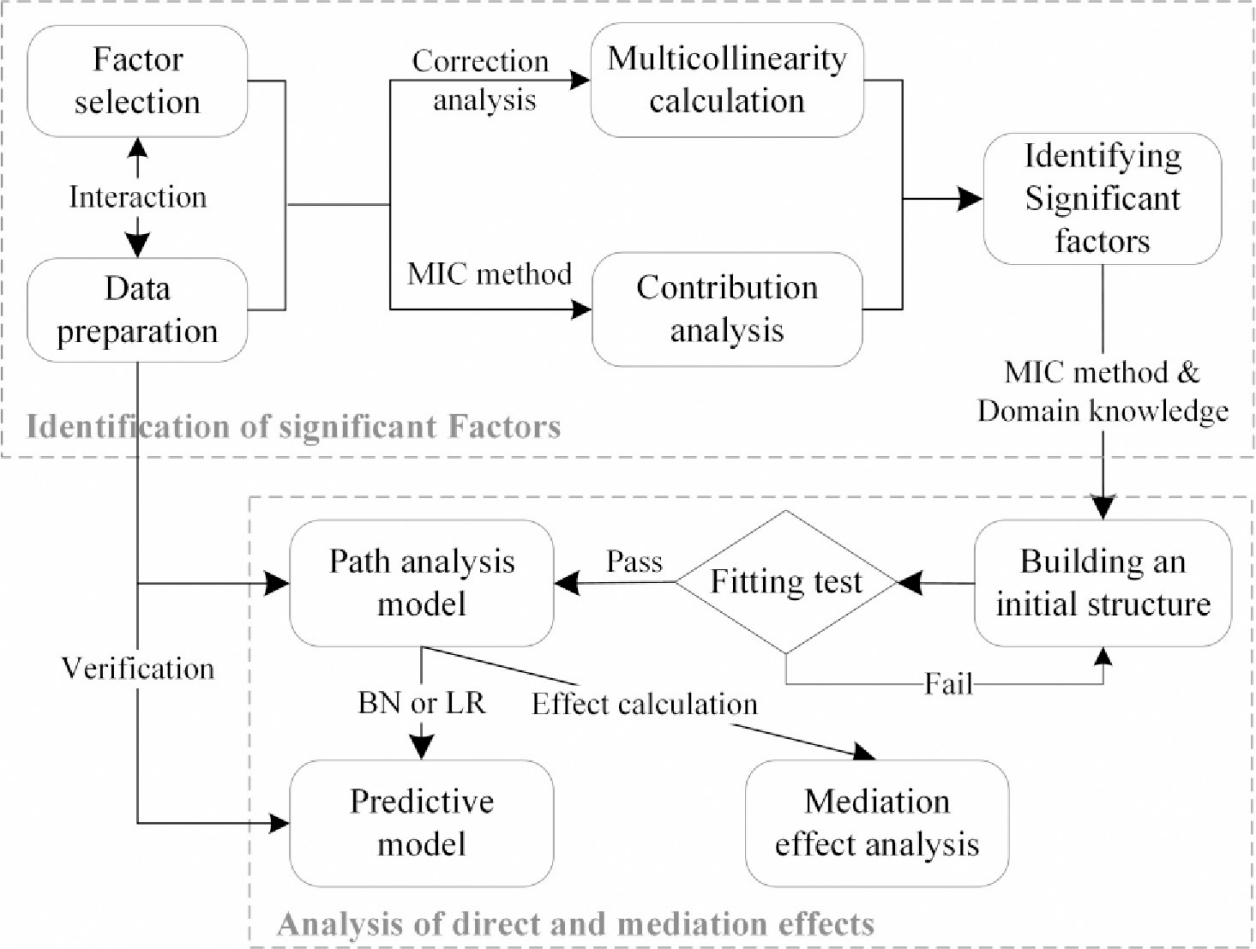
The flow chart for identifying the key factors and constructing a path analysis model in this study.

Path analysis is a combination of multiple regression equations that can analyse the causal relationships between factors, as well as their direct and indirect effects on LP, and obtain more accurate causal contributions. However, because path analysis needs to determine the causal relationships by assumptions in advance, it is subjective, and assumption errors will cause the model to be revised multiple times, which requires much work to finalize the model structure. Therefore, this paper studies how to identify the key factors of seismic liquefaction and uses the path analysis method to analyse their direct and mediation effects on LP without a correlation hypothesis. The research idea is shown in Fig 1. First, because of the lack of subjective assumptions about factor relationships in the path analysis method, based on the collected data and factors, on the one hand, the correlation analysis method is used to eliminate variables with multicollinearity; on the other hand, the maximum information coefficient (MIC) method is used to quantitatively screen out the relatively important variables and determine their nonlinear relationships. Then, domain knowledge is used to determine the direction of causal influence and obtain an initial path structure, which can greatly reduce the number of manual adjustments to the model structure. Finally, the significance and multiple measurement indexes are used to verify the fitting effect of the initial structure. When the fit is not good, the links between factors can be appropriately added to improve the performance of the model and obtain revised impact path models until the final model passes the test. After an analysis of the direct and mediation effects of the key factors on LP, their comprehensive contributions can be further identified. In addition, the causal model can directly provide the structure of a Bayesian network (BN) model for parameter learning, or it can also be directly extracted as a logistic regression (LR) model for predicting liquefaction. The performances of these two models are verified through the collected data.

## Methodology

### Correlation analysis method

Correlation analysis is generally used to describe the relationship and multicollinearity between two variables. For different variable types, the calculation equations are different. For instance, the Pearson correlation coefficient [10] is used to quantitatively describe the relational degree between two continuous variables that conform to the normal distribution; the Spearman correlation coefficient [11] is used to quantitatively describe the rank correlation between any continuous variable and an ordinal variable, and the Kendall correlation coefficient [11] is used to quantitatively describe the contingency relation between two categorical variables or between any continuous variable and a categorical variable. Their calculation functions are as follows:

$$\rho_{Pearson}=\mathrm{cov}\left(x,y\right)/\left(\sigma_{x}\sigma_{y}\right)$$ (1)

$$\rho_{Spearman}=\mathrm{cov}\left(rg_{x},rg_{y}\right)/\left(\sigma_{rgx}\sigma_{rgy}\right)$$ (2)

$$\rho_{Kendall}=\left(n_{c}-n_{d}\right)/\left[0.5n\left(n-1\right)\right]$$ (3)

where *ρ*_*Pearson*_, *ρ*_*Spearmas*_ and *ρ*_*Kendall*_ are the Pearson, Spearman, and Kendall correlation coefficients, respectively; cov(*x*,*y*) is the covariance of variables *x* and *y*; *σ*_*x*_ and *σ*_*y*_ are the standard deviations of *x* and *y*; *rg*_*x*_ and *rg*_*y*_ stand for the rank transformed values of *x* and *y*; *n* is the sample size; *n*_*c*_ and *n*_*d*_ are the numbers of concordant and discordant variables in *x* and *y*, respectively. The coefficient values range from -1.0 to 1.0. A correlation coefficient of -1.0 shows a perfect negative correlation, while a correlation coefficient of 1.0 denotes a perfect positive correlation. If a correlation coefficient value between the two variables is larger than or equal to 0.9, it means they exhibit multicollinearity.

Since the above correlation analysis methods do not perform well when calculating the nonlinear correlation between two variables, Reshef et al. [12] proposed a measuring method, the maximum information coefficient (MIC), for the dependence of two-variable relationships. The MIC is based on the idea that if a relationship exists between two variables, then a grid can be drawn on the scatterplot of the two variables that partitions the data to encapsulate that relationship. Thus, the largest possible mutual information can be calculated for every pair of integers (x, y) based on mutual information theory. After normalizing these mutual information values, the highest normalized mutual information is the MIC value. More details can be found in Reshef et al. [12]. The MIC calculated equation is as follows:

$$MIC\left(x,y\right)=\underset{x\times y<B\left(n\right)}{\max}\frac{\max\left\{I\left(x,y\right)\right\}}{\log_{2}\left(\min\left\{x,y\right\}\right)}=\underset{x\times y<B\left(n\right)}{\max}\frac{\max\sum^{i}\sum^{j}P\left(x_{i},y_{j}\right)\log_{2}P\left(x_{i},y_{j}\right)/\left\{P\left(x_{i}\right)P\left(y_{j}\right)\right\}}{\log_{2}\left(\min\left\{x,y\right\}\right)}$$ (4)

where *I*(*x*,*y*) is the mutual information of variables *x* and *y* in a grid; *i* and *j* are the line and column numbers of the grid, respectively; *n* is the sample size; *x*×*y*<*B*(*n*) denotes the boundary of the grid; normally, *B*(*n*) = *n*^0.6^; *P*(*x*_*i*_) and *P*(*y*_*i*_) are the frequency of occurrence of *x*_*i*_ and *y*_*i*_ in a small square given a grid, respectively; *P*(*x*_*i*_,*y*_*j*_) is the joint probability density of the two variables that is equal to the frequency of simultaneous occurrence of *x*_*i*_ and *y*_*i*_ in a small square. Normally, if *MIC*(*x*,*y*)≥0.9Max*MIC*(*X*) or *MIC*(*y*,*x*)≥0.9Max*MIC*(*Y*), *x* and *y* are correlated. Thus, the MIC method can obtain most of the correct connections among variables [13]. Max*MIC*(*X*) and Max*MIC*(*Y*) are the maxima in a given row and column, respectively. In addition, if *MIC*(*x*_1_,*y*) is much less than the others *MIC*(*x*_*i*_,*y*) (i ≠ 1), *x*_*1*_ produces little impact on *y*.

### Path analysis method

**Fig 2:**
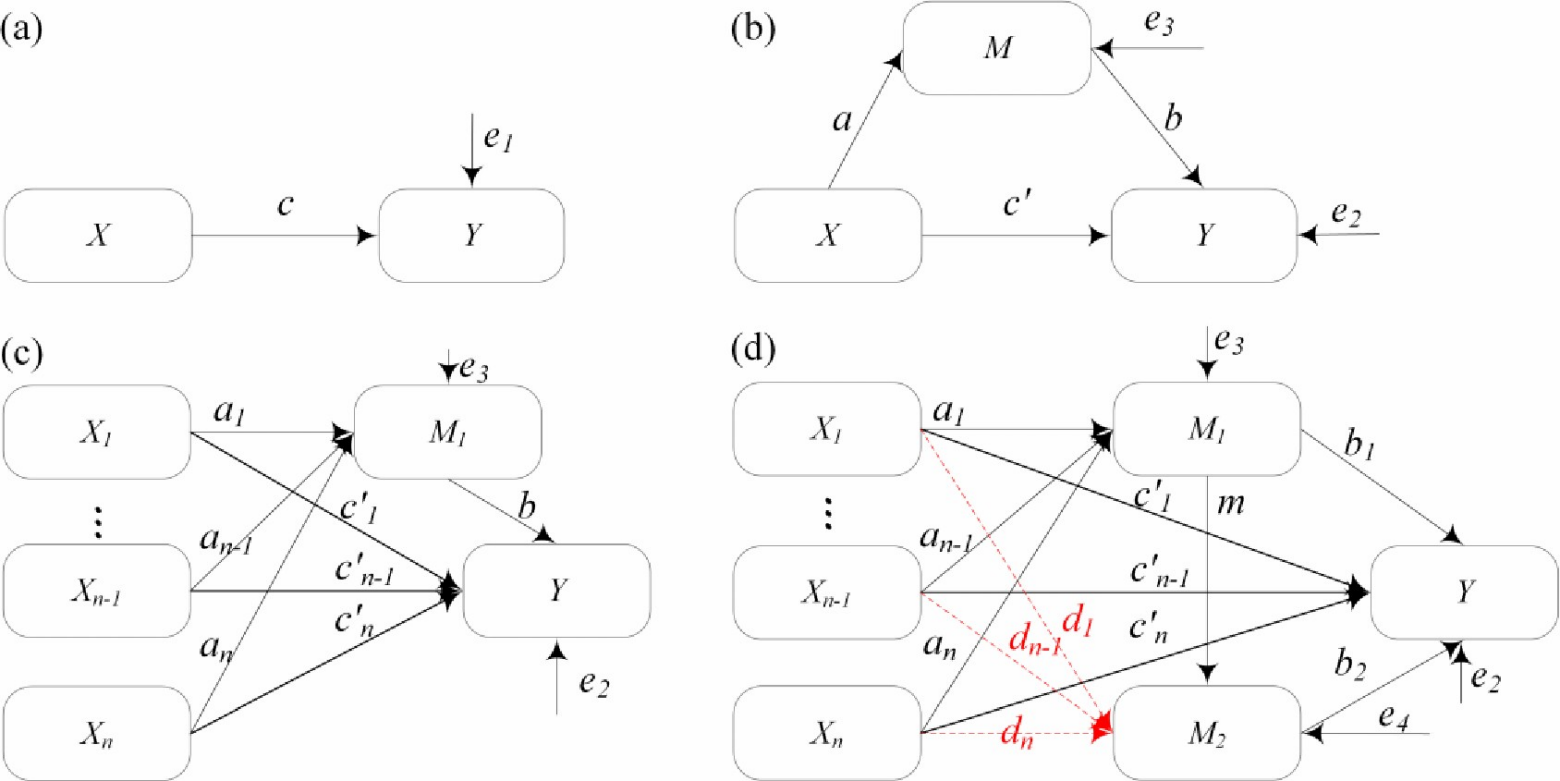
Mediation effect models: (a) a total effect model; (b) a simple mediation model; (c) a single-step multiple mediation model; (d) a multiple-step multiple mediation model.

Path analysis is a method of causality analysis first proposed by Wright [14]. The path diagram (see Fig 2) can help researchers clearly understand the influence path between variables (arrow direction) and the degree and properties of causal influence (the magnitude and positiveness of the coefficient) and analyse the direct, mediation, and total effects of independent variables on the dependent variables. The path analysis method has been widely used in the fields of psychology, sociology, and economics but less in the field of civil engineering. To date, the path analysis method has not been applied in seismic liquefaction analysis. Since path analysis does not contain latent variables, it is a special case of structural equation modelling. Path analysis includes the following four steps:

1. Assumptions about the causal relationships between variables.
2. Collection of enough data and calculation of the path coefficient. Kline [15] recommended that the sample size should be 10 times (or ideally 20 times) the number of parameters. The calculation of path coefficients is designed to solve the regression coefficients of multiple regression equations, which can usually be calculated by special softwares, such as SPSS, Amos, Mplus, etc.
3. Inspection and revision of the model. The estimated values of the regression coefficients need to be tested for statistical significance and the critical proportion value of the C.R. If the coefficients are not statistically significant (normally larger than 0.05) or the absolute value of the C.R. is less than 1.96, the above steps should be repeated, that is, redefine the assumptions and calculate the path coefficients, until the significance and the C.R. value of the model meet the requirements. After the above test is passed, the goodness of fit of the model needs to be examined using multiple statistical fit indexes. If the test fails, the model needs to be manually corrected, such as by adding some links, to improve the goodness of fit of the model.
4. Effects analysis. The researchers can determine the direct effect and the mediation effect of any independent variable on the dependent variable. For example, in Fig 2B, the direct effect is *c*’, the mediation effect is *a*⋅*b*, and its total effect is *c*’+*a*⋅*b*. It is worth noting that path analysis is a technique for testing causality but cannot be used to discover or search for causality.

The statistical fit indexes include absolute indexes, comparative indexes, and parsimonious indexes for the goodness of fit, where the absolute indexes contain the ratio of likelihood-ratio *χ*^2^ values to degrees of freedom values (*χ*^2^/*df*), root mean square error of approximation (RMSEA), the goodness of fit index (GIF), and adjusted goodness of fit index (AGIF); the comparative indexes contain the comparative fit index (CFI), normed fit index (NFI), relative fit index (RFI), incremental fit index (IFI), and Tucker-Lewis fit index (TLI); the parsimonious indexes contain the parsimony goodness of fit index (PGFI), parsimony normed fit index (PNFI), and parsimony-adjusted comparative fit index (PCFI). The calculation equations for all of these indexes and their standard values for indicating a well-fitted model (shown in Table 2) can be found in the references [15–18]. Generally, it is difficult for a model to meet the requirements of all fit indexes. Therefore, as long as most indexes can meet their standard ranges, then the model possesses a good fit. In addition, the smaller the values of the Akaike information criteria (AIC), Bayesian information criteria (BIC), and Browne-Cudeck criterion (BCC) are, the better the model fit.

**Table 2:** Factors and their influencing rules for earthquake-induced liquefaction.

Statistical fit index	Absolute index					Comparative index				Parsimonious index		
	χ^2^/*df*	P-value	*RMSEA*	*GFI*	*AGFI*	*NFI*	*IFI*	*TLI*	*CFI*	*PGFI*	*PNFI*	*PCFI*
Standard value	< 5	< 0.05	< 0.08	> 0.9	> 0.9	> 0.9	> 0.9	> 0.9	> 0.9	> 0.5	> 0.5	> 0.5

### Mediation effect

The mediation effect is mainly used to study the influence path and mechanism of the independent variable acting on the dependent variable indirectly through the mediation variable. Fig 2B shows a simple mediation model. In addition to the independent variable *X* directly affecting the dependent variable *Y*, it can also affect *Y* through a variable *M*. Thus, *M* is considered to play a mediating role between *X* and *Y*, and it is called the mediator. In Fig 2A, however, *X* produces only a direct effect on *Y* but not a mediation effect. If there is a mediation effect on the influence of *X* on *Y*, but the influence is not considered, it is unable to fully explain the influence of *X* on *Y*.

In most studies of mediation effect models, when the independent variable, mediator, and dependent variable are all continuous variables, linear regression analysis can be used directly to construct a model. However, there are relatively few studies on the situation where the dependent variable is a binary variable, such as the occurrence of seismic liquefaction. A common approach is to use logistic regression instead of linear regression in the analysis of the independent variables and dependent variables, as well as mediation analysis [19]. The calculation equations are as follows:

$$M=\beta_{3}+aX+e_{3}$$ (5)

$$Y'=LogitP\left(Y=1|X\right)=\ln\frac{P\left(Y=1|X\right)}{P\left(Y=0|X\right)}=\beta_{1}+cX+e_{1}$$ (6)

$$Y''=LogitP\left(Y=1|M,X\right)=\ln\frac{P\left(Y=1|M,X\right)}{P\left(Y=0|M,X\right)}=\beta_{2}+c'X+bM+e_{2}$$ (7)

where *M* is a mediator; *X* is an independent variable; and *Y* is a binary dependent variable (*Y* = 0 or 1). *a*, *b*, *c* and *c*’ are the fitting parameters or regression coefficients in the regression analysis, where *a* denotes the influence of *X* on *M*; *b* denotes the influence of *M* on *Y*; *c* and *c*’ denote the direct influences of *X* on *Y* with and without considering the influence of *M*, respectively. P(*Y*|*X*) and P(*Y*|*M*,*X*) are the conditional probabilities of *Y* given *X* and *M*, respectively. *e*_1_ and *e*_2_ are the residuals of *Y* in the model (a) and model (b), respectively; *e*_3_ are the residuals of *M*. *β*_1_, *β*_2_, and *β*_3_ are regression constant terms in Eqs (6), (7) and (5), respectively.

In Fig 2B, there are generally two methods for calculating the size of the mediation effect; one is the coefficient difference method, i.e., *c*−*c*’; another is the coefficient product method, i.e., *a*⋅*b*. MacKinnon et al. [19] found that *a*⋅*b* is closer to the true value of the mediation effect, and compared with *c*−*c*’, it has good robustness and can better represent the mediation effect. Therefore, *a*⋅*b* is used to represent the mediation effect in this study. However, the units of *b*, *c* and *c*’ in logistic regression are logits, and they are inconsistent with *a* of the linear regression in scale. In addition, *c* and *c*’ of Eqs (6) and (7), respectively, are also different in scale due to their different independent variables. Thus, one cannot simply multiply *a* and *b*. To solve the problem of different scales for the different regression equations, MacKinnon and Dwyer [20] proposed an approach to standardize regression coefficients. The calculation equations are as follows:

$$a^{std}=a\cdot SD\left(X\right)/SD\left(M\right)$$ (8)

$$b^{std}=b\cdot SD\left(M\right)/SD\left(Y''\right)=b\cdot SD\left(M\right)/\sqrt{c{'}^{2}\mathrm{var}\left(X\right)+b^{2}\mathrm{var}\left(M\right)+2c'b\cdot \mathrm{cov}\left(X,M\right)+\pi^{2}/3}$$ (9)

$$c^{std}=c\cdot SD\left(X\right)/SD\left(Y'\right)=c\cdot SD\left(X\right)/\sqrt{c^{2}\mathrm{var}\left(X\right)+\pi^{2}/3}$$ (10)

$$c{'}^{std}=c'\cdot SD\left(X\right)/SD\left(Y''\right)=c'\cdot SD\left(X\right)/\sqrt{c{'}^{2}\mathrm{var}\left(X\right)+b^{2}\mathrm{var}\left(M\right)+2c'b\mathrm{cov}\left(X,M\right)+\pi^{2}/3}$$ (11)

where the *std* superscript denotes the standardization of logistic regression coefficients. *SD*(⋅) is the standard deviation of a variable; var(⋅) is the variance of a variable; cov(*X*,*M*) is the covariance of *X* and *M*. Thus, the mediation effect of *X* is changed to *a*^*std*^*b*^*std*^. The total effect is equal to the sum of the direct effect and the mediation effect, i.e., *c*’^*std*^+*a*^*std*^*b*^*std*^. When *c*’^*std*^ and *a*^*std*^*b*^*std*^ possess the same sign, the mediation effect is complementary, and the mediation effect ratio is *a*^*std*^*b*^*std*^/(*c*’^*std*^+*a*^*std*^*b*^*std*^). However, if their signs are different, e.g., *c*’^*std*^ is positive whereas *a*^*std*^*b*^*std*^ is negative, the mediation effect is competitive, i.e., the suppression effect is present by MacKinnon et al. [21]. The suppression effect ratio is |*a*^*std*^*b*^*std*^/*c*’^*std*^|.

Since the mediation effect model contains a binary dependent variable, and its mediation effect equals, *Z*_*a*_×*Z*_*b*_, this study uses the Sobel method suggested by Iacobucci [22] to test the significance of the product of coefficients *a*^*std*^*b*^*std*^. The calculation equations are as follows:

$$Z=a^{std}b^{std}/SE\left(a^{std}b^{std}\right)=a^{std}b^{std}/\sqrt{{\left(a^{std}\right)}^{2}{\left(SE\left(b^{std}\right)\right)}^{2}+{\left(b^{std}\right)}^{2}{\left(SE\left(a^{std}\right)\right)}^{2}}$$ (12)

$$SE\left(a^{std}\right)=SE\left(a\right)SD\left(X\right)/SD\left(M\right)$$ (13)

$$SE\left(b^{std}\right)=SE\left(b\right)SD\left(M\right)/SD\left(Y''\right)$$ (14)

where *SE*(⋅) denotes the standard error of the regression coefficient; a |*Z*| value larger than 1.96 indicates that the indirect effect of *X* on *Y* is significant; otherwise, there is no mediation effect.

When there are multiple independent variables and mediators, the model becomes very complicated, as shown in Fig 2C and 2D. Fig 2C is a single-step multiple mediation model, and Fig 2D is a multiple-step multiple mediation model [23]. In Fig 2D, in addition to the direct effects of the independent variable *X*_1_,*X*_2_,⋯,*X*_*n*_ on the dependent variable *Y*, there are two parallel mediation effects via *M*_1_ and *M*_2_ and a chain mediation effect from *M*_1_ to *M*_2_. Thus, the regression equations are as follows:

$$M_{1}=\beta_{3}+\sum_{i=1}^{n}a_{i}X_{i}+e_{3}$$ (15)

$$M_{2}=\beta_{4}+mM_{1}+\sum_{i=1}^{n}d_{i}X_{i}+e_{4}$$ (16)

Y''={LogitP(Y=1|M1,Xi)=lnP(Y=1|M1,Xi)P(Y=0|M1,Xi)=β2+∑i=1nci'Xi+b1M1+e2forFig.2(c)LogitP(Y=1|M1,M2,Xi)=lnP(Y=1|M1,M2,Xi)P(Y=0|M1,M2,Xi)=β2+∑i=1nci'Xi+∑j=12bjMj+e2forFig.2(d) (17)

where *n* is the number of independent variables; *i* = 1,2,⋯,*n*; *j* = 1,2; *M*_1_ and *M*_2_ are mediators; *e*_4_ are the residuals of *M*_2_; *β*_4_ is the regression constant term in Eq (16). The total effects of any variable in Fig 2C and 2D are equal to $c_{i}^{'}{}^{{}^{std}}+a_{i}^{std}b^{std}$ and $c_{i}^{'}{}^{{}^{std}}+a_{i}^{std}b_{1}^{std}+d_{i}^{std}b_{2}^{std}+a_{i}^{std}m^{std}b_{2}^{std}$, respectively, and their mediation effects are $a_{i}^{std}b^{std}$ and $a_{i}^{std}b_{1}^{std}+d_{i}^{std}b_{2}^{std}+a_{i}^{std}m^{std}b_{2}^{std}$, respectively. For multiple mediation effects in Fig 2D, there are three terms with one for the specific mediation effect (e.g., $a_{i}^{std}b_{1}^{std}$, $d_{i}^{std}b_{2}^{std}$ or $a_{i}^{std}m^{std}b_{2}^{std}$), one for the total mediation effect (e.g., $a_{i}^{std}b_{1}^{std}+d_{i}^{std}b_{2}^{std}+a_{i}^{std}m^{std}b_{2}^{std}$) and one for the contrast mediation effect (e.g., $a_{i}^{std}m^{std}b_{2}^{std}-d_{i}^{std}b_{2}^{std}$, $a_{i}^{std}b_{1}^{std}-d_{i}^{std}b_{2}^{std}$ or $a_{i}^{std}m^{std}b_{2}^{std}-a_{i}^{std}b_{1}^{std}$) [23]. The specific mediation effect ratio is equal to the specific mediation effect divided by the sum of the absolute values of each specific mediation effect, i.e., $\left|a_{i}^{std}b_{1}^{std}\right|/\left(\left|a_{i}^{std}b_{1}^{std}\right|+\left|d_{i}^{std}b_{2}^{std}\right|+\left|a_{i}^{std}m^{std}b_{2}^{std}\right|\right)$. Similar to the mediation effect ratio in Fig 2B, if the direct effect $c_{i}^{'}{}^{{}^{std}}$ and total mediation effect possess the same sign, the mediation effect ratio is $\left(a_{i}^{std}b_{1}^{std}+d_{i}^{std}b_{2}^{std}+a_{i}^{std}m^{std}b_{2}^{std}\right)/\left(c_{i}^{'}{}^{{}^{std}}+a_{i}^{std}b_{1}^{std}+d_{i}^{std}b_{2}^{std}+a_{i}^{std}m^{std}b_{2}^{std}\right)$. However, if their signs are opposite, their suppression effect ratio is $\left|\left(a_{i}^{std}b_{1}^{std}+d_{i}^{std}b_{2}^{std}+a_{i}^{std}m^{std}b_{2}^{std}\right)/c_{i}^{'}{}^{{}^{std}}\right|$. In addition, the *Z* test for $a_{i}^{std}m^{std}b_{2}^{std}$ is changed to:

$$\begin{array}{l} Z=a_{i}^{std}m^{std}b_{2}^{std}/SE\left(a_{i}^{std}m^{std}b_{2}^{std}\right)\\ \;=\frac{a_{i}^{std}m^{std}b_{2}^{std}}{\sqrt{{\left(a_{i}^{std}\right)}^{2}{\left(SE\left(m^{std}\right)\cdot SE\left(b_{2}^{std}\right)\right)}^{2}+{\left(m^{std}\right)}^{2}{\left(SE\left(a_{i}^{std}\right)\cdot SE\left(b_{2}^{std}\right)\right)}^{2}+{\left(b_{2}^{std}\right)}^{2}{\left(SE\left(a_{i}^{std}\right)\cdot SE\left(m^{std}\right)\right)}^{2}}} \end{array}$$ (18)

when there are more than two mediators, and readers can derive this equation by themselves according to the formula suggested by Sobel [24]. *SD*(*Y*") for calculating *SE*(*d*^*std*^) and *SE*(*b*^*std*^) can be expressed by

$$SD\left(Y''\right)=\sqrt{\begin{array}{l} \sum_{i=1}^{n}c{'}_{i}^{2}\mathrm{var}\left(X_{i}\right)+\sum_{j=1}^{2}b_{j}^{2}\mathrm{var}\left(M_{j}\right)+2\sum_{i=1}^{n}\sum_{j=1}^{2}c{'}_{i}b_{j}\mathrm{cov}\left(X_{i},M_{j}\right)\\ +2\sum_{i=1}^{n}\sum_{k=1,i\neq k}^{n}c{'}_{i}c{'}_{k}\mathrm{cov}\left(X_{i},X_{k}\right)+\pi^{2}/3 \end{array}}$$ (19)

## The historical case data

Many factors affect earthquake-induced liquefaction, and these factors are summarized in Table 1. However, some factors are difficult to characterize or quantify with a certain indicator (e.g., particle shape, soil structure, etc.) or their values are difficult to obtain in the historical database (e.g., permeability coefficient, liquid-plastic limit index, particle size distribution). Therefore, 19 factors, as shown in Table 3, are initially selected in this study based on these two principles and in consideration of the limitations of the data sources. These factors are the *M*_*w*_, *R*, *PGA*, *t*, *I*, *FC*, *D*_*50*_, *ST*, *V*_*s*_ (shear wave velocity), *V*_*s1*_ (the overburden stress-corrected shear wave velocity), *D*_*w*_, *D*_*s*_, *H*_*n*_, *D*_*n*_, *σ*_*V*_, $\sigma_{V}^{'}$, *T*_*s*_, *DT*, and *A*, where *V*_*s1*_ is the correction value of *V*_*s*_ considering the effect of $\sigma_{V}^{'}$, and it can characterize the relative density of the critical layer [25]. The 659 data are collected from 40 historical earthquakes, of which the earliest is the 1906 San Francisco earthquake and the most recent is the 2011 Christchurch earthquake. Of the 659 cases, 29 cases were removed because of missing data. In the remaining 630 cases, 51 are from Japan, 185 are from America, 253 are from China (including Taiwan), 94 are from New Zealand, and 47 are from other locations in the world. The sample size is larger than 20 times the number of the parameters estimated in the path analysis model (that is, 29 in Fig 8), or at least 200 cases [15], which can ensure the validity of parameter fitting in the path analysis.

**Table 3:** Statistical characteristics of the cases.

Variable	Mean & variance	Range	Sample ratio	Variable	Mean & variance	Range	Sample ratio
*M*_*w*_	7.05 0.48	4.5 < *M*_*w*_ < 6	6.5%	*D*_*w*_ (m)	2.03 1.923	0 ≤ *D*_*w*_ < 1	20.8%
		6 ≤ *M*_*w*_ < 7	40.5%			1 ≤ *D*_*w*_ < 2	36.3%
		7 ≤ *M*_*w*_ < 8	50.3%			2 ≤ *D*_*w*_ < 3	24.9%
		8 ≤ *M*_*w*_	2.7%			3 ≤ *D*_*w*_	17.9%
*R* (km)	47.74 1281.51	0 < *R* ≤ 10	23.5%	*D*_*s*_ (m)	5.53 8.25	0 ≤ *D*_*s*_ < 3	14.8%
		10 < *R* ≤ 50	35.1%			3 ≤ *D*_*s*_ < 5	36.2%
		50 < *R* ≤ 100	32.4%			5 ≤ *D*_*s*_ < 10	40.3%
		100 < *R*	9.0%			10 ≤ *D*_*s*_	8.7%
*t* (s)	28.50 626.31	0 < *t* ≤ 10	17.5%	$\sigma_{V}^{'}$ (kPa)	67.69 1011.34	0 < $\sigma_{v}^{'}$ < 30	4.9%
		10 < *t* ≤ 30	45.4%			30 ≤ $\sigma_{v}^{'}$ < 50	30.5%
		30 < *t* ≤ 60	26.3%			50 ≤ $\sigma_{v}^{'}$ < 100	48.7%
		60 < *t*	10.8%			100 ≤ $\sigma_{v}^{'}$	15.9%
*PGA* (g)	0.28 0.024	0 ≤ *PGA* < 0.15	14.8%	*σ*_*V*_ (kPa)	102.85 2827.78	0 < *σ*_*V*_ < 60	17.5%
		0.15 ≤ *PGA* < 0.3	45.4%			60 ≤ *σ*_*V*_ < 100	40.8%
		0.3 ≤ *PGA* < 0.4	13.8%			100 ≤ *σ*_*V*_ < 200	26.8%
		0.4 ≤ *PGA*	26.0%			200 ≤ *σ*_*V*_	14.9%
*I*	7.45 0.893	*I* ≤ 6	8.4%	*T*_*s*_ (m)	3.59 5.74	0 < *T*_*s*_ < 2	23.5%
		*I* = 7	42.7%			2 ≤ *T*_*s*_ < 4	45.2%
		*I* = 8	38.4%			4 ≤ *T*_*s*_ < 6	20.5%
		9 ≤ *I*	10.5%			6 ≤ *T*_*s*_	10.8%
*D*_*50*_ (mm)	1.36 12.54	*D*_*50*_ ≤ 0.075	8.7%	*D*_*n*_ (m)	0.79 1.12	*D*_*n*_ = 0	49.8%
		0.075 < *D*_*50*_ ≤ 0.25	59.7%			0 < *D*_*n*_ ≤ 1	18.3%
		0.25 < *D*_*50*_ ≤ 2	14.4%			1 < *D*_*n*_ ≤ 2	17.5%
		2 < *D*_*50*_	17.1%			2 < *D*_*n*_	14.4%
*FC* (%)	19.88 485.86	0 < *FC* < 5	36.2%	*H*_*n*_ (m)	1.88 2.88	*H*_*n*_ = 0	29.4%
		5 ≤ *FC* < 15	24.4%			0 < *H*_*n*_ ≤ 1	9.2%
		15 ≤ *FC* < 35	18.9%			1 < *H*_*n*_ ≤ 2	20.2%
		35 ≤ *FC* < 70	14.9%			2 < *H*_*n*_ ≤ 4	30.3%
		70 ≤ *FC*	5.6%			4 < *H*_*n*_	11.0%
*V*_*s*_ (m/s)	158.02 2175.27	*V*_*s*_ ≤ 120	18.9%	*ST*	-	Silty clay to clayey silt	5.6%
		120 < *V*_*s*_ ≤ 140	20.6%			Silt to sand mixtures	13.3%
		140 < *V*_*s*_ ≤ 160	19.7%			Sandy silt to silty sand	17.5%
		160 < *V*_*s*_ ≤ 200	26.0%			Sand mixture to sand	19.0%
		200 < *V*_*s*_	14.8%			Clean sand (FC < 5%)	21.9%
*V*_*s1*_ (m/s)	177.22 2017.51	*V*_*s1*_ ≤ 140	17.0%			Gravel mixture to gravel	2.9%
		140 < *V*_*s1*_ ≤ 160	21.9%			Gravel and gravelly sand	19.8%
		160 < *V*_*s1*_ ≤ 175	16.2%	*DT*	-	Fill	1.6%
		175 < *V*_*s1*_ ≤ 210	26.0%			Fill, hydraulic	6.2%
		210 < *V*_*s1*_	18.9%			Fill, dumped	2.2%
*A*	-	Recent	18.7%			Fill, uncompacted	1.1%
		Holocene	70.6%			Fill, improved	1.0%
		Pleistocene	10.6%			Alluvial	35.1%
*LP*	-	0	33.5%			Alluvial, fluvial	52.5%
		1	66.5%			Volcanic debris flow	0.3%

For each case, the site behaviour is characterized through a binary indicator LP, where LP = 1 if liquefaction occurred and LP = 0 if it did not occur, and the surveyed fields are limited to level and gently sloping sites. Table 3 shows the statistical characteristics of the cases. Almost every variable possesses an uneven proportion between groups, especially for LP; the liquefied sample size is approximately twice that of the non-liquefied sample size, and there is sampling bias, which affects the performance of the liquefaction prediction model [26] but does not affect the parameter estimation of the path analysis model. The collected data cover almost all possible liquefaction situations, such as *M*_*w*_ between 5 and 9.2, *PGA* between 0.1 and 0.789 g, *FC* between 0% and 99%, *D*_*50*_ between 0.006 and 33.4 mm, *V*_*s*_ between 59 and 380 m/s, *D*_*w*_ between 0 and 7 m, *D*_*s*_ between 1.1 and 17.8 m, etc., which facilitates the construction of a reliable causal model.

## Construction of a multiple causal path model

### Identification of the key factors

To avoid the adverse effects of multicollinearity on the performance of the model and to further identify variables that produce less impact on liquefaction, this section first uses the Pearson, Spearman, and Kendall methods to calculate the correlations between factors and find variables with correlations greater than 0.9. Then, the MIC method is used to calculate the nonlinear relationship between factors and liquefaction and to identify the factors with the largest contributions.

**Fig 3:**
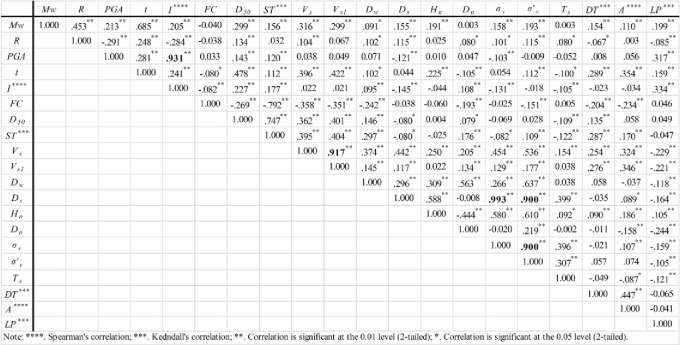
Correlation coefficients of factors.

Fig 3 shows the correlations of the selected variables. The Kendall correlation coefficient between *PGA* and *I* and the Pearson correlation coefficients between *V*_*s*_ and *V*_*s1*_, *D*_*s*_ and *σ*_*V*_, and *D*_*s*_ and $\sigma_{V}^{'}$ are larger than or equal to 0.9, so there are multicollinearities among them. Between *PGA* and *I*, *I* should be eliminated because it is a subjective variable, and it is difficult to establish a physical connection with the occurrence of liquefaction. Between *V*_*s1*_ and *V*_*s*_, *V*_*s1*_ should be removed because *V*_*s1*_ is a correction of the *V*_*s*_ value considering the effect of $\sigma_{V}^{'}$, so there would be a compound effect of *V*_*s1*_ on liquefaction if it is not removed. Between *D*_*s*_, *σ*_*V*_ and $\sigma_{V}^{'}$, $\sigma_{V}^{'}$ contains the effect of *D*_*w*_ on liquefaction, whereas *D*_*s*_ is a conventional variable that is easier to obtain than the other two variables. Therefore, *σ*_*V*_ and $\sigma_{V}^{'}$ are removed. Thus, 15 factors are kept for further identification of their significance using the MIC method.

**Fig 4:**
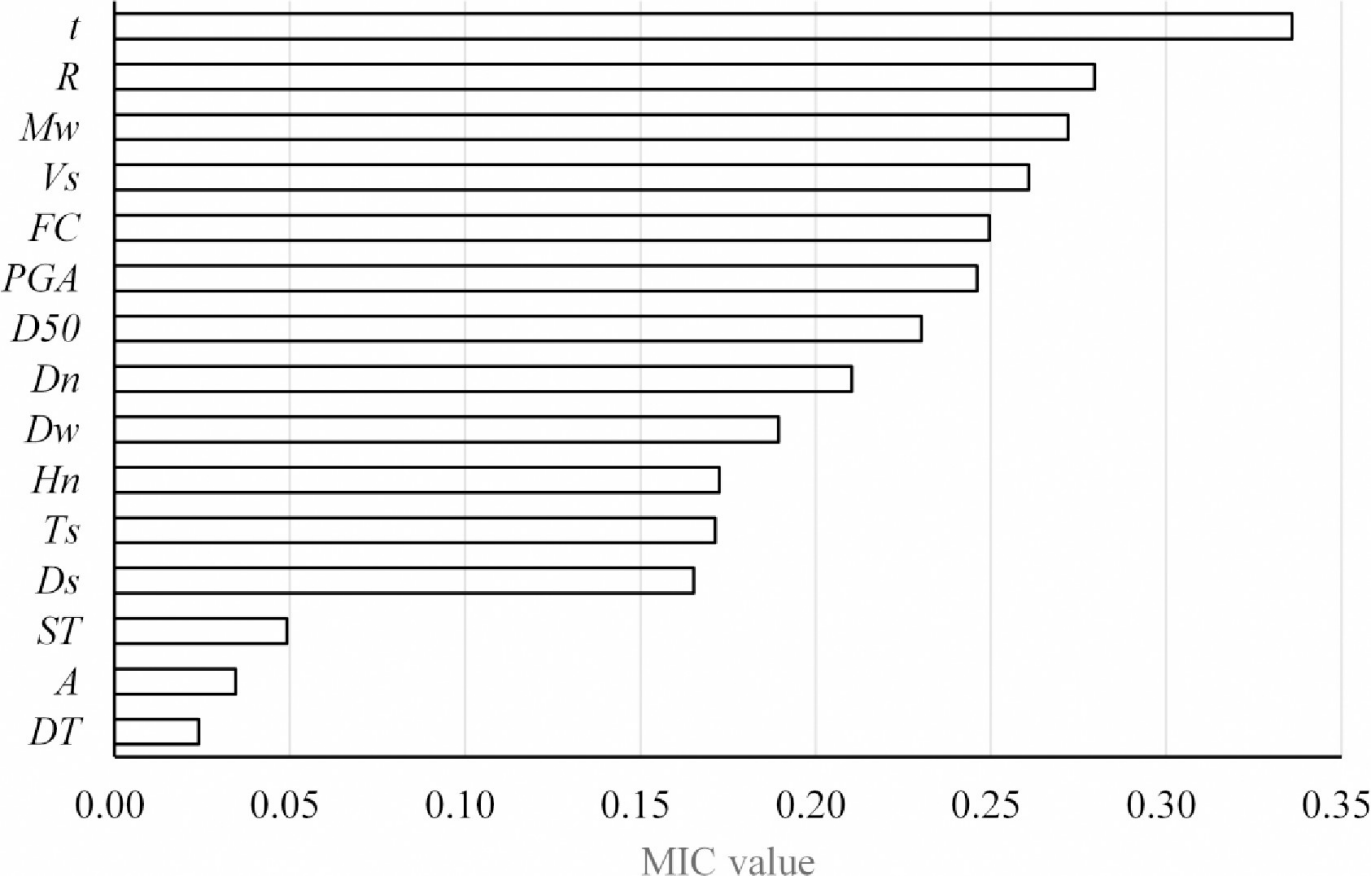
The MIC values between factors and LP.

Fig 4 shows the MIC values of the 15 factors for LP. The MIC values of *ST*, *DT*, and *A* are much smaller than those of other factors. Therefore, *t*, *R*, *M*_*w*_, *V*_*s*_, *FC*, *PGA*, *D*_*50*_, *D*_*n*_, *D*_*w*_, *H*_*n*_, *T*_*s*_, and *D*_*s*_ are considered the key factors. It should be noted that the *I*, *V*_*s1*_, *σ*_*V*_ and $\sigma_{V}^{'}$ factors that were excluded in the multicollinearity analysis are not insensitive to LP. Their MIC values are 0.13, 0.20, 0.24, and 0.28, respectively, which shows that they are also key factors. They were ignored only because of multicollinearity.

**Fig 5:**
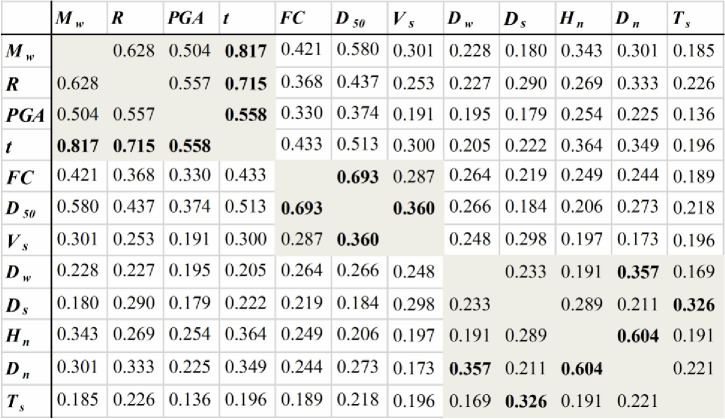
The MIC values between the 12 key factors.

**Fig 6:**
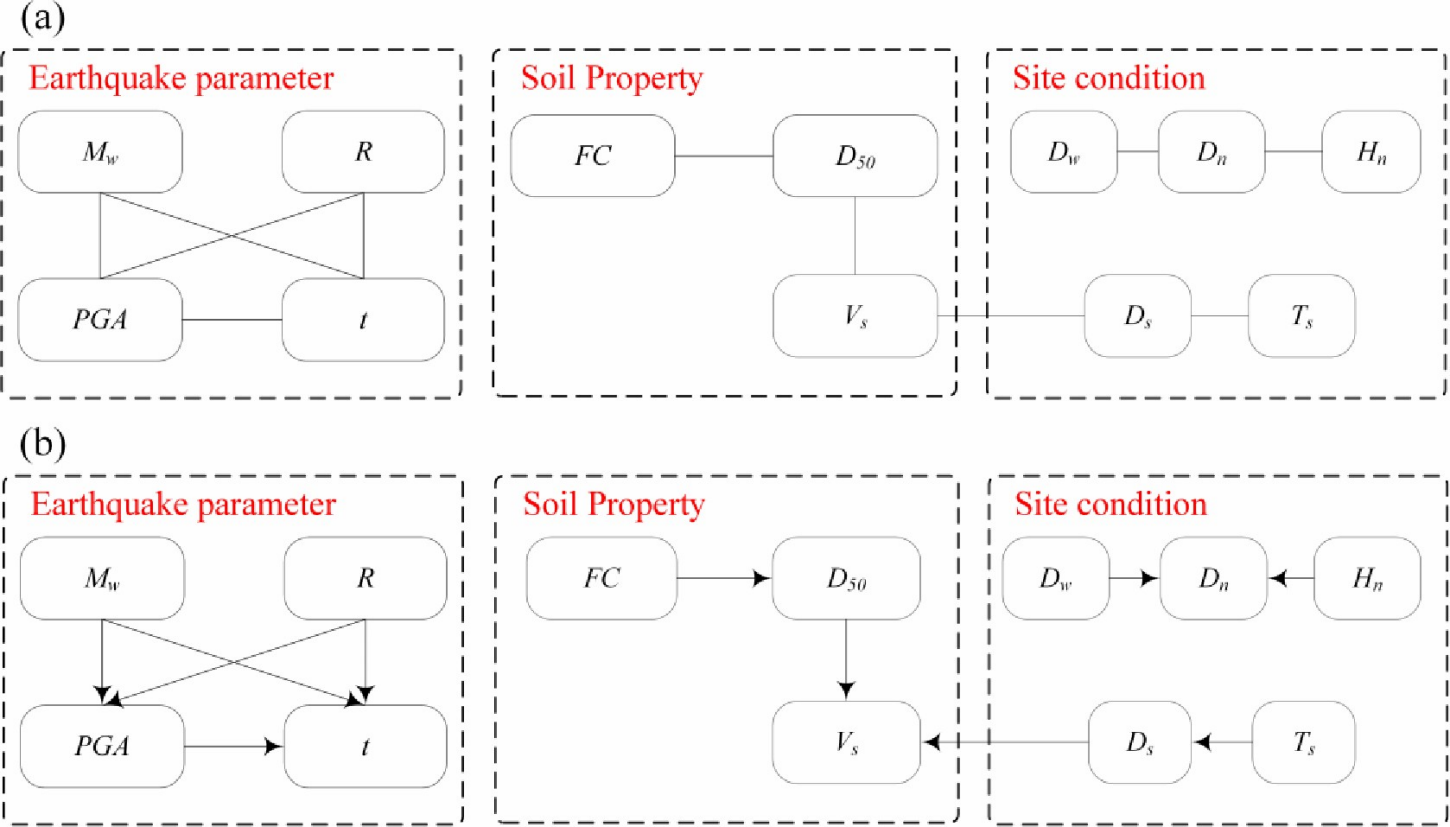
Links between factors: (a) relational structures; (b) causal structures.

Fig 5 shows the MIC values between the 12 key factors and LP. The variables whose MIC values are greater than 0.9 times the maximum MIC value of the rows or columns are *MIC*(*M*_*w*_, *t*), *MIC*(*M*_*w*_, *PGA*), *MIC*(*R*, *t*), *MIC*(*R*, *PGA*), *MIC*(*PGA*, *t*), *MIC*(*FC*, *D*_50_), *MIC*(*V*_*s*_, *D*_50_), *MIC*(*D*_*w*_, *D*_*n*_), *MIC*(*D*_*s*_,*V*_*s*_), *MIC*(*H*_*n*_, *D*_*n*_), and *MIC*(*T*_*s*_, *D*_*s*_). Therefore, there are links between them, as shown in Fig 6A. It can be seen that the relationship between the variables is not directional because the MIC method can only identify the nonlinear correlation between variables. To obtain the causalities between variables, domain knowledge as shown in Table 1 is used to determine the causal direction of the variables in this study. For example, for the same site, the larger the *M*_*w*_ is, the larger the *PGA*, and so the *M*_*w*_ affects the *PGA*, not *PGA* affects *M*_*w*_. Using domain knowledge to determine the causal direction is very simple and convenient. When the research problem does not include domain knowledge, mathematical methods can be used to calculate the causal direction [13]. In particular, there is no direct physical relationship between *FC* and *D*_*50*_, but usually *D*_*50*_ decreases as *FC* increases. In contrast, however, the relationship may not be true. Therefore, this study assumes that *FC* is the cause of *D*_*50*_. The causal model is determined as shown in Fig 6B, and the direction of the arrow indicates cause and effect.

### Construction of an initial path model and its correction

Generally, the path analysis method is used for analysing linear causality between variables. However, most of the factors of seismic liquefaction exhibit nonlinear relationships. Therefore, this paper has computed the natural logarithms of some variables according to their functional forms (as shown in Table 4) and converted them into a linear equation in the path analysis. Moreover, the processed variables also approximately follow the normal distribution.

**Table 4:** Functional relationships between some variables.

Functional relationship	Reference
ln*Y* = *a*+*b*⋅*M*_*w*_+*c*ln*R*	[27]
ln*D*_50_ = *a*+*b*⋅*FC*	[28]
ln*V*_*s*_ = *a*+*b*⋅*D*_*s*_	This study
*D*_*n*_ = *D*_*w*_−*H*_*n*_ (when *D*_*n*_ is negative, *D*_*n*_ = 0)	[5]

Note: *a*, *b*, and *c* are estimated parameters; *Y* is an earthquake parameter such as *PGA* or *t*.

**Fig 7:**
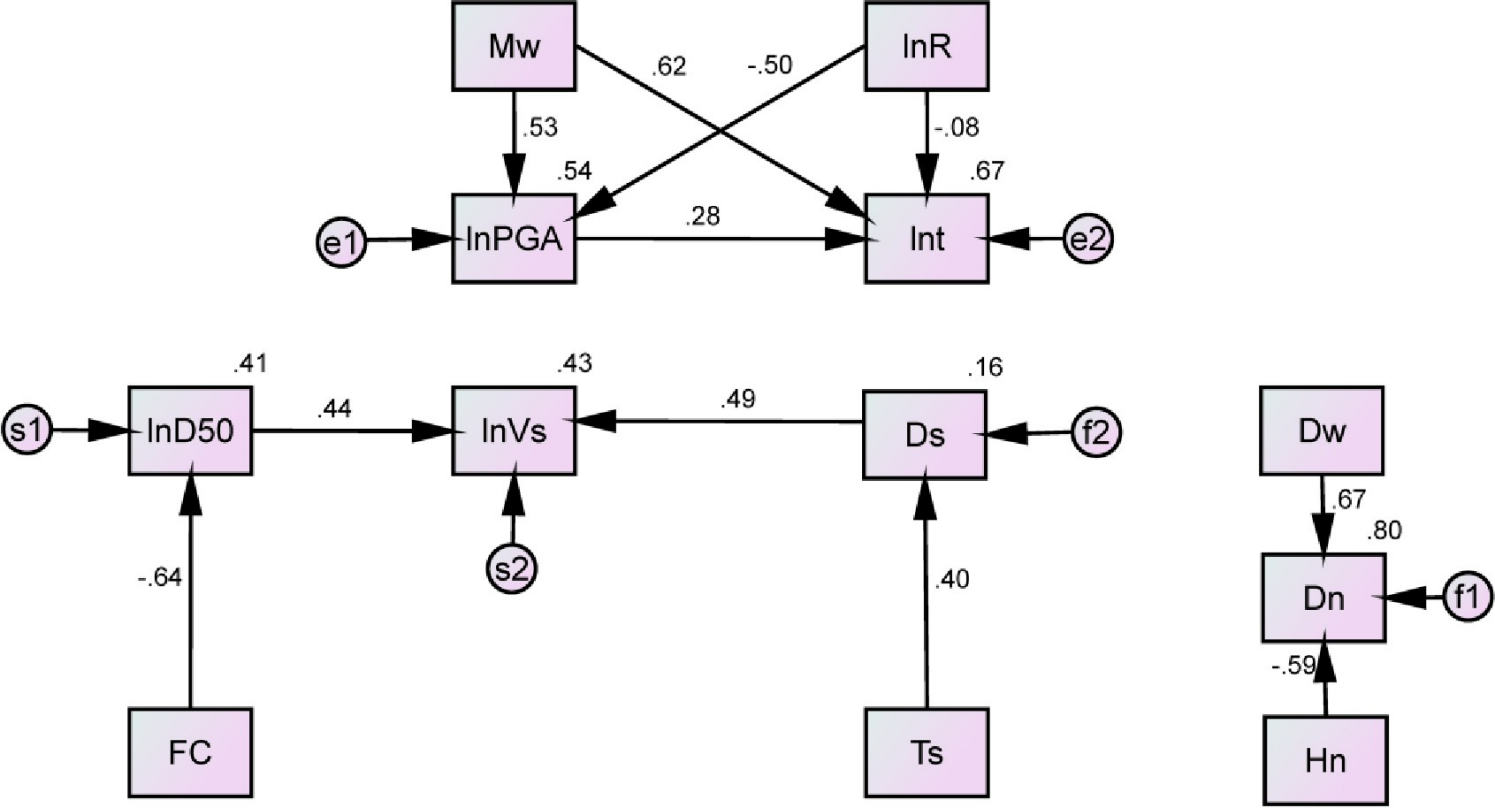
An initial path analysis model with standardized estimates.

An initial path analysis model, as shown in Fig 7, is constructed according to the causal structure in Fig 6. The values on arrows in Fig 7 are the standardized path coefficients, and the values in the upper right corner of the variables are regression coefficients of determination of dependent variables. The path coefficients and statistical indexes in Fig 7 are calculated with the Amos software (Version 27), as shown in Table 5. It can be seen that the C.R. values are greater than 1.96, and the P-values are less than 0.05. Therefore, the causality path constructed by the MIC method combined with domain knowledge is effective. However, except for the parsimonious indexes, other statistical fit indexes almost fall short of the standard values. Therefore, it is necessary to add some new links in the initial model, recalculate the path coefficients, and evaluate the fit indexes.

**Table 5:** The initial model with the paths and their statistical test indexes.

Path	Unstandardized coefficient	S.E.	C.R.	P-value	Statistical fit index
*M*_*w*_ → *PGA*	0.576	0.029	19.630	***	Absolute indexes:
*M*_*w*_ → *t*	0.906	0.043	21.223	***	χ^2^/*df* = 20.914; P-value = 0.000; RMSE = 0.166
*R* → *PGA*	-0.420	0.023	-18.512	***	GFI = 0.801; AGFI = 0.717
*R* → *t*	-0.095	0.032	-2.940	0.003	Comparative indexes:
*PGA* → *t*	0.378	0.046	8.268	***	NFI = 0.689; IFI = 0.699
*D*_*50*_ → *V*_*s*_	0.081	0.005	14.787	***	TLI = 0.638; CFI = 0.697
*FC* → *D*_*50*_	-0.046	0.002	53.959	***	Parsimonious indexes:
*D*_*w*_ → *D*_*n*_	0.590	0.016	37.962	***	PGFI = 0.565; PNFI = 0.574; PCFI = 0.582
*H*_*n*_ → *D*_*n*_	-0.426	0.013	-33.532	***	Information indexes:
*T*_*s*_ → *D*_*s*_	0.478	0.044	10.904	***	AIC = 1196.285; BIC = 1298.510;
*D*_*s*_ → *V*_*s*_	0.049	0.003	16.281	***	BCC = 1197.229

Note: S.E. means standard error of estimated parameter; C.R. means the absolute values of the critical ratio

*** means the P-value less than 0.001.

**Fig 8:**
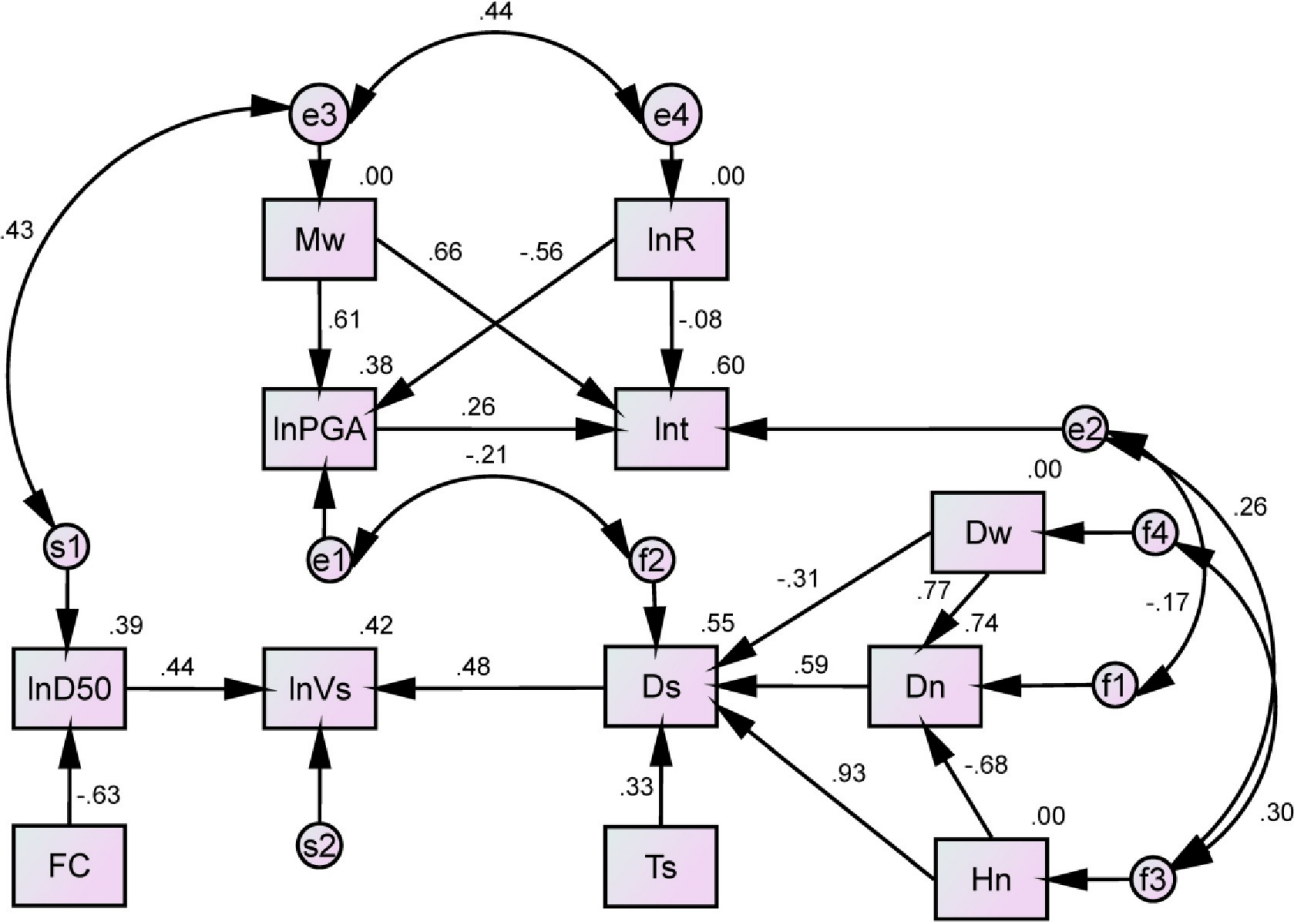
A modified path analysis model with standardized estimates.

According to the modification indexes (MI) for improving the performance of the model, the links between variables corresponding to the large MI values are added to the initial model. The revised model is shown in Fig 8. Compared with Figs 7 and 8 adds three new links between variables (i.e., links between *D*_*s*_ and *D*_*w*_, *D*_*s*_ and *D*_*n*_, and *D*_*s*_ and *H*_*n*_) and six correlations (e.g. correlation coefficient 0.44 between two residual terms of *M*_*w*_ and *R*) between the residual terms. The correlations between the residual terms may be caused by the exclusion of some factors, or they may show that these variables are mathematically correlated. This issue requires further study in the future. However, adding the correlations of the residual terms does not affect the path causalities of the model. After recalculating the path coefficients, it is found that all the statistical indexes of the path coefficients are significant as shown in Table 6, and most of the model’s fitness indexes pass the test except for χ^2^/df, RMSE, and AGFI, but the values of these three indexes are close to their standard values. In addition, compared with the initial model, the values of the information indexes in the modified model are largely decreased. Therefore, the fitting effect of the improved model is acceptable, and it is appropriate for an analysis of the effects.

**Table 6:** The modified model with paths and their statistical test indexes.

Path	Unstandardized coefficient	S.E.	C.R.	P-value	Statistical fit index
*M*_*w*_ → *PGA*	0.569	0.032	17.851	***	Absolute indexes:
*M*_*w*_ → *t*	0.897	0.043	20.351	***	χ^2^/*df* = 5.926; P-value = 0.000; RMSE = 0.088
*R* → *PGA*	-0.405	0.025	-16.365	***	GFI = 0.937; AGFI = 0.893
*R* → *t*	-0.087	0.033	-2.668	0.008	Comparative indexes:
*PGA* → *t*	0.378	0.043	8.723	***	NFI = 0.926; IFI = 0.938;
*D*_*50*_ → *V*_*s*_	0.081	0.006	14.582	***	TLI = 0.910; CFI = 0.938
*FC* → *D*_*50*_	-0.045	0.002	23.073	***	Parsimonious indexes:
*D*_*w*_ → *D*_*n*_	0.592	0.016	36.700	***	PGFI = 0.553; PNFI = 0.646; PCFI = 0.653
*H*_*n*_ → *D*_*n*_	-0.426	0.013	-31.895	***	Information indexes:
*T*_*s*_ → *D*_*s*_	0.384	0.031	12.522	***	AIC = 336.609; BIC = 478.872;
*D*_*s*_ → *V*_*s*_	0.049	0.003	15.846	***	BCC = 337.959
*D*_*w*_ → *D*_*s*_	-0.629	0.098	-6.442	***	
*H*_*n*_ → *D*_*s*_	1.535	0.074	20.884	***	
*D*_*n*_ → *D*_*s*_	1.561	0.136	11.508	***	

### Construction of a multiple casual path model for liquefaction

**Fig 9:**
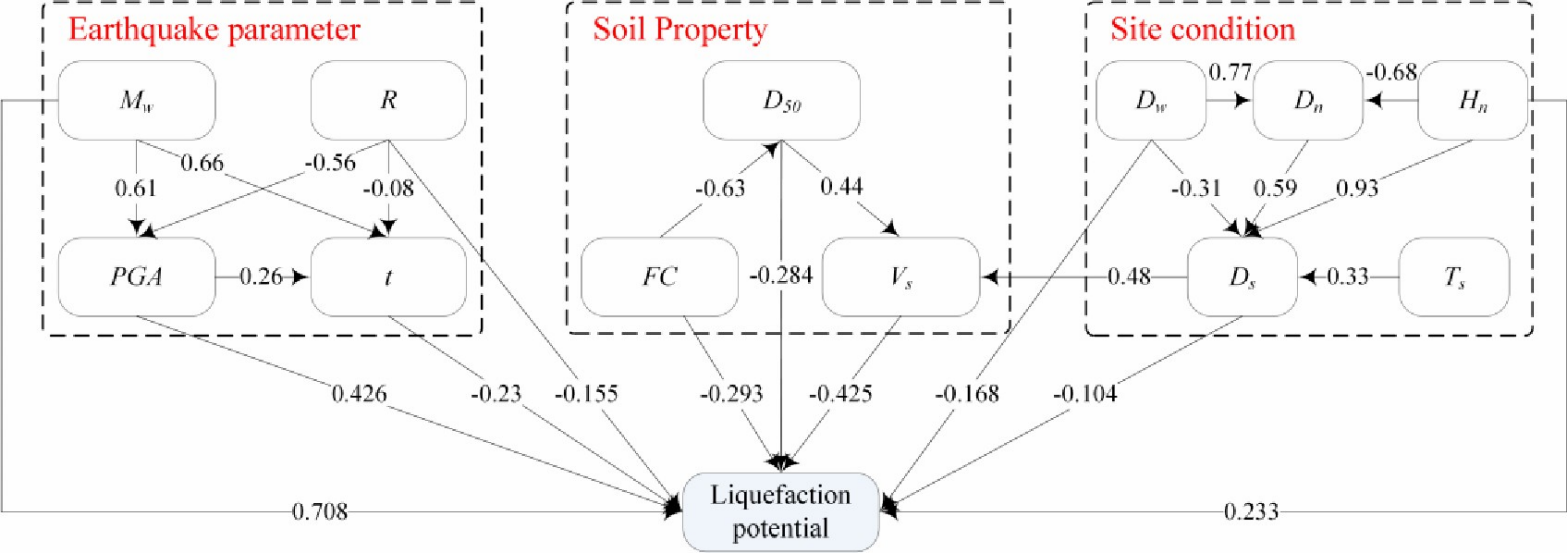
A multiple mediation model of earthquake-induced liquefaction with standardized estimates.

In the above section, the path model of the factors of liquefaction was constructed. In this study, LP is treated as a binary variable, and it cannot be directly analysed with the Amos software along with its factors. Therefore, a stepwise logistic regression method is first adopted to construct a model between LP and its factors and eliminate some links with insignificant effects on LP. For example, the coefficients of *T*_*s*_ and *D*_*n*_ do not pass the significance test, so they possess no direct links to LP. However, their influences on liquefaction can be produced indirectly through *D*_*s*_. Then, after combining the LR model and the modified model, a multiple mediation model of seismic liquefaction can be constructed, as shown in Fig 9. The multiple mediation model is also a recursive causal model because it can not only reflect the influences of the factors on liquefaction but also the interactions between factors. The logistic regression function and path functions are as follows:

$$P_{L}=1/\left[1+\exp\left(\begin{array}{l} 3.406M_{w}-0.576\ln R+2.169\ln PGA-0.816\ln t-0.044FC\\ -0.593\ln D_{50}-4.901\ln V_{s}-0.402D_{w}-0.12D_{s}+0.454H_{n}+10.159 \end{array}\right)\right]$$ (20)

$$\ln PGA=0.576M_{w}-0.42\ln R-4.013$$ (21)

$$\ln t=0.906M_{w}-0.095\ln R+0.378\ln PGA-2.512$$ (22)

$$\ln V_{s}=0.049D_{s}+0.081\ln D_{50}+4.846$$ (23)

$$\ln D_{50}=-0.046FC-0.305$$ (24)

$$D_{s}=1.565H_{n}+1.608D_{n}-0.695D_{w}+0.393T_{s}+1.324$$ (25)

$$D_{n}=0.59D_{w}-0.426H_{n}+0.392$$ (26)

where *P*_*L*_ is the probability of LP; all estimates in the regression functions are significant.

## Results

### Analysis of direct and total effects of the factors on liquefaction

**Fig 10:**
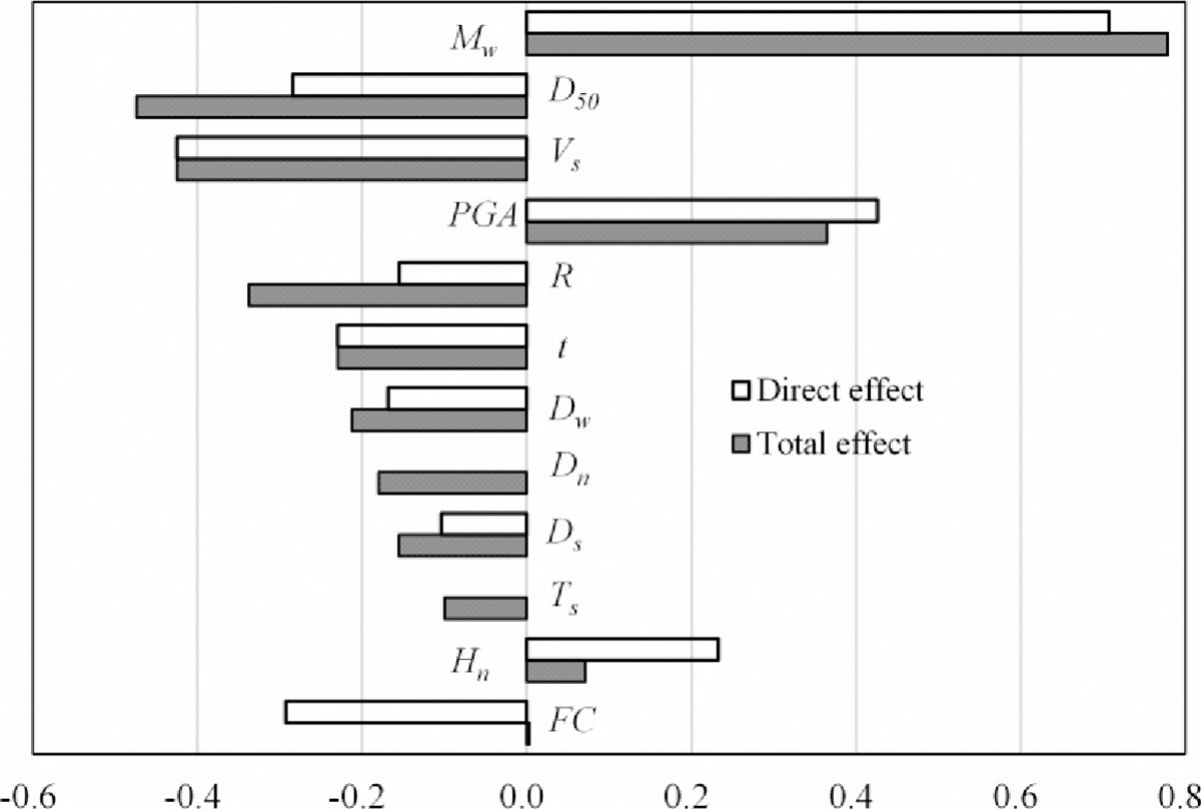
The direct and total effects of the factors on liquefaction.

Fig 10 shows the direct and total effects of the factors on liquefaction. It can be seen that there is a large difference between the direct effect and total effect of some factors, e.g., the total effects of *D*_*n*_ and *T*_*s*_ are -0.18 and -0.1 (a negative sign represents inhibition), respectively, whereas their direct effects are zero; the direct effects of *H*_*n*_ and *FC* are 0.233 and -0.293, respectively, whereas their total effects are 0.072 (a positive sign represents promotion) and 0.003, respectively. Therefore, only considering the direct effects of factors (i.e., the regression coefficients in the LR model) and ignoring their mediation effects leads to large sensitivity deviations of the factors in the analysis of significant contributions.

For the total effects of factors, *M*_*w*_, *PGA*, *FC*, and *H*_*n*_ induce positive effects on liquefaction, whereas *D*_*50*_, *V*_*s*_, *R*, *t*, *D*_*w*_, *D*_*n*_, *D*_*s*_, and *T*_*s*_ induce negative effects on liquefaction. The results are close to the influence rules in Table 1 except for *H*_*n*_ and *t*, which will be discussed in Section 6. The absolute values of the total effects of these factors are ranked as *M*_*w*_, *D*_*50*_, *V*_*s*_, *PGA*, *R*, *t*, *D*_*w*_, *D*_*n*_, *D*_*s*_, *T*_*s*_, *H*_*n*_, and *FC* in descending order, which is different from the order of MIC values between the factors and LP, especially the ranking of *FC*. This is because the relationships between these factors (mediation effects) are not considered when calculating the MIC values. However, when the mediation effect is not considered, the rankings of the direct effects and MIC values are not much different. In addition, comparing the direct or total effects of earthquake parameters, soil properties, and site conditions, the effects of earthquake parameters (*M*_*w*_, *PGA*, *t*, and *R*) are much larger than those of the other two terms for most factors. These findings are consistent with the conclusions found in the literature [9].

### Analysis of multiple mediation effects

Table 7 shows the multiple mediation effects of the factors on liquefaction. It can be seen that all mediation paths pass the *Z* test because their absolute values are larger than 1.96. For all factors except *t* and *V*_*s*_, their influences on liquefaction include at least one mediation path, e.g., the mediation effect of *R* on LP not only through *PGA* or *t* (*R* → *PGA* → *LP* or *R* → *t* → *LP*) but also through *PGA* to *t* (*R* → *PGA* → *t* → *LP*), which forms multiple chain mediation effects. For factors with multiple mediation effects, the sizes and signs of their specific mediation effects are different. For instance, the specific mediation effect of *R* → *PGA* → *LP* is equal to -0.236 (a negative value means suppression), whereas the specific mediation effect of *R* → *t* → *LP* is equal to 0.019 (a positive value means promotion), and the ratio of its specific mediation effect is much less than that of the path *R* → *PGA* → *LP*. Therefore, the mediation effect of *PGA* as a mediation variable is much stronger than that of *t*; i.e., for *R*, *PGA* is more important than *t* for predicting liquefaction.

**Table 7:** The multiple mediation effects of the factors on liquefaction.

Mediation path	|*Z*| value	Specific mediation effect	The ratio of the specific mediation effect	Total mediation effect	Mediation effect ratio	suppression effect ratio
*R* → *PGA* → *LP*	6.03	-0.236	81.8%	-0.183	54.2%	-
*R* → *t* → *LP*	2.07	0.019	6.6%			
*R* → *PGA* → *t* → *LP*	25.42	0.034	11.6%			
*M*_*w*_ → *PGA* → *LP*	6.10	0.256	58.0%	0.071	9.1%	-
*M*_*w*_ → *t* → *LP*	3.28	-0.149	33.7%			
*M*_*w*_ → *PGA* → *t* → *LP*	25.99	-0.036	8.3%			
*PGA* → *t* → *LP*	3.13	0.061	100.0%	0.061	16.6%	-
*D*_*50*_ → *V*_*s*_ → *LP*	6.36	-0.190	100.0%	-0.190	40.1%	-
*FC* → *D*_*50*_ → *LP*	4.50	0.178	59.9%	0.296	-	101.2%
*FC* → *D*_*50*_ → *V*_*s*_ → *LP*	80.45	0.119	40.1%			
*D*_*s*_ → *V*_*s*_ → *LP*	2.09	-0.051	100.0%	-0.051	32.9%	-
*T*_*s*_ → *D*_*s*_ → *LP*	2.08	-0.033	33.3%	-0.100	100%	-
*T*_*s*_ → *D*_*s*_ → *V*_*s*_ → *LP*	67.11	-0.067	66.7%			
*D*_*n*_ → *D*_*s*_ → *LP*	2.07	-0.060	33.3%	-0.180	100%	-
*D*_*n*_ → *D*_*s*_ → *V*_*s*_ → *LP*	63.66	-0.120	66.7%			
*H*_*n*_ → *D*_*s*_ → *LP*	2.10	-0.094	23.2%	-0.161	-	69.0%
*H*_*n*_ → *D*_*s*_ → *V*_*s*_ → *LP*	89.23	-0.189	46.5%			
*H*_*n*_ → *D*_*n*_ → *D*_*s*_ → *LP*	22.76	0.041	10.1%			
*H*_*n*_ → *D*_*n*_ → *D*_*s*_ → *V*_*s*_ → *LP*	1134.09	0.082	20.2%			
*D*_*w*_ → *D*_*s*_ → *LP*	2.00	0.032	13.5%	-0.044	20.7%	-
*D*_*w*_ → *D*_*s*_ → *V*_*s*_ → *LP*	4.79	0.063	27.1%			
*D*_*w*_ → *D*_*n*_ → *D*_*s*_ → *LP*	2.10	0.046	19.8%			
*D*_*w*_ → *D*_*n*_ → *D*_*s*_ →*V*_*s*_ → *LP*	1171.06	0.093	39.6%			

In addition, mediation effects include indirect-only mediation effects (e.g., *T*_*s*_ and *D*_*n*_ with mediation effect ratios of 100%) and partial mediation effects (e.g., *R*, *M*_*w*_, *PGA*, *D*_*50*_, *D*_*s*_, and *D*_*w*_). Comparing these mediation effect ratios, the mediation effects of *R*, *T*_*s*_, and *D*_*n*_ are greater than their direct effects. If their mediation effects are ignored when analysing their importance, the results will be biased. Moreover, there are two factors, *FC* and *H*_*n*_, that_,_ produce suppressive effects. When analysing their influences on liquefaction, in addition to their mediation effects, their suppression effects should also be considered. For example, the suppression effect ratio of *FC* is as high as 101.2%; that is, the suppression effect is greater than the absolute of the direct effect, which reverses its influence on liquefaction, and this mechanism is consistent with the influence rule of *FC* in Table 1. Therefore, analysing the mediation and covering the effects of factors is helpful for further understanding of the mechanism of liquefaction. In addition to *FC* and *H*_*n*_, *T*_*s*_ may exhibit a suppression effect, but *T*_*s*_ is considered to have no direct effect in the causal model, so it is considered an indirect-only mediator. This situation is related to the collected data and requires the collection of more data for verification or updating.

### Predictive performance of the causal model

In the construction of the causal path analysis model, it can be found that the model can directly extract a liquefied LR prediction model such as Eq (20). The logistic regression model with an accuracy of 84.8% (73.9% and 90.2% for non-liquefaction and liquefaction cases, respectively) in its training performance shows a strong learning ability. To further analyse the predictive performance of the model, 5-fold cross-validation is used to train and test the model by equally dividing the collected data into 5 folds [29]. In the crossover trial, four folds are used for training the model, and the remaining fold is used for testing its predictive performance. The process is repeated 5 times so that each fold is involved in training and testing. In addition, the causal path model can be directly taken as a structure of the BN model; discretization of factors according to Table 3, and parameter learning based on the divided data are conducted to learn the parameters or conditional probabilities of the model using the expectation-maximization algorithm. The detail of parameter learning can refer to Hu and Liu [29]. The 5-fold cross-validation is used to verify its performance.

**Fig 11:**
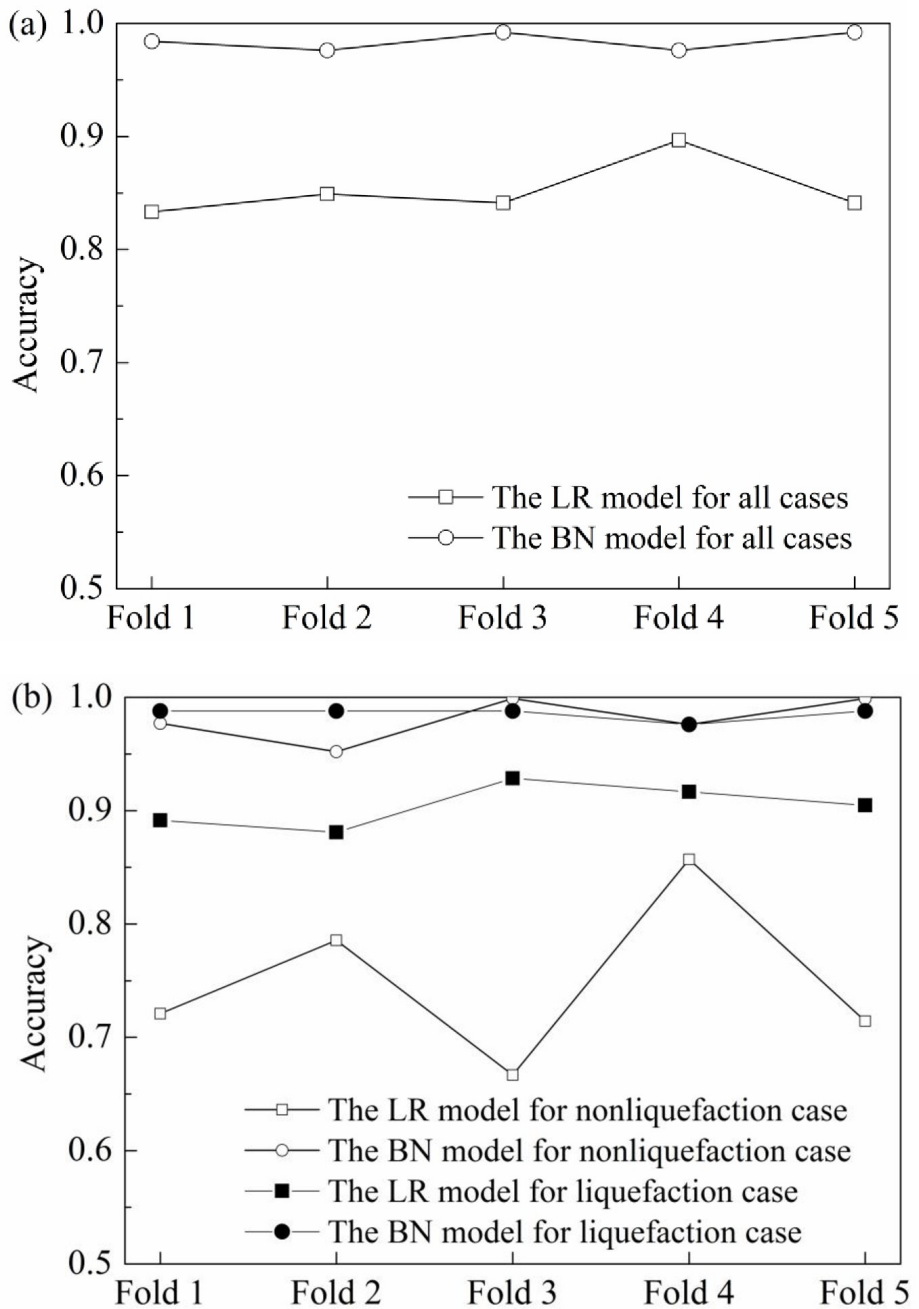
Comparisons of the performances of the LR and BN models in the 5-fold validation test: (a) Accuracy for each fold dataset; (b) Accuracy for liquefaction and non-liquefaction cases in each fold dataset.

In the 5-fold cross-validation, the comparisons of the performances of the LR and BN models are shown in Fig 11. It can be seen that the accuracies of the BN model are better than those of the LR model in each fold test, as well as in the prediction of liquefied and non-liquefied cases in each fold dataset. The reason is that the LR model ignores the impact of the important factors on liquefaction, e.g. *D*_*n*_, whereas the BN model contains the impact of the factor, as well as other factors, e.g. *T*_*s*_. In addition, the parameters in the LR model are constant, whereas parameters in the BN model are taken as random variables, their values are probability distributions that are more suitable for the calculation of uncertain problems such as liquefaction prediction. What’s more, it is worth noting that these two models are more capable of identifying liquefied samples than non-liquefied samples. This is because the liquefied sample size in this study is larger than the non-liquefied sample size, i.e., there is a sampling bias in the training process for each model. Hu et al. [26] suggested that the best sampling bias ratio is between 1 and 1.5 (liquefaction/non-liquefaction) for the BN model and approximately 0.5 for the LR model. However, the ratio of liquefied samples to non-liquefied samples is approximately 2 beyond the recommended ranges. This issue can be dealt with using the oversampling technique or adding more data to balance the ratio [26].

## Discussion

This study proposes an approach to quantify the importance of factors and uses a multiple mediation effects model to prove that many factors of liquefaction not only produce direct effects but also significant mediation effects. The 12 key factors identified in this study are almost the same as those concluded in Tang et al. [2], except for grain composition, drainage conditions such as permeability coefficient, and OCR. For the unselected factors, such as the permeability coefficient, grain composition, soil structure, etc., if their MIC values are large, they will be identified as significant factors and vice versa. Furthermore, these factors will slightly affect the structure of the causal model in Fig 9 due to adding new variables to the model, but will not affect the mediation effects of other factors, and might slightly increase the total effects of relevant factors. However, they are not selected because they are difficult to obtain in the historical database. Thus, if the data of these factors are available, their effects should also be considered in the path analysis. In addition, it is worth noting that several variables that were eliminated due to multicollinearity, namely, *I*, *σ*_*V*_, $\sigma_{V}^{'}$, and *V*_*s1*_, are also key factors. Therefore, when selecting important factors for predicting seismic liquefaction, these factors should be considered as candidates according to engineering demands.

The multiple mediation model constructed in this study can not only analyse the direct effects of the factors on liquefaction but also their mediation effects and suppression effects. Therefore, it can effectively avoid serious evaluation biases regarding contributions of the factors on liquefaction like the LR model that can only analyze the direct effects of factors. In addition, the causal model can also compare the mediation effects of different paths such as *R* → *PGA* → *LP* and *R* → *t* → *LP*, which is helpful for a clearer understanding of the liquefaction mechanism of multi-factor coupling. However, because the causal model ignores the influences of site conditions on seismic parameters, this may cause a certain deviation of the indirect influence of seismic parameters on liquefaction in the causal model.

Comparing the total effects of factors in the causal model and the correlation coefficients in Fig 3, it can be found that most factors exhibit the same influence characteristics on liquefaction except for *t* and *D*_*50*_. Obtaining a different or “wrong” sign in these two methods is a common phenomenon [30]. For example, *t* produces a negative effect on LP in the casual model, whereas it produces a positive effect in the correlation analysis. This is because the correlation analysis only considers the correlation between *t* and LP, while the regression analysis can consider both the effect of *t* on LP and the effects of other variables related to *t* on LP. When the inhibiting effects of other variables are too large, the regression coefficients exhibit anti-regular phenomena. McGuire and Barnhard [31] and Trifunac and Brady [32] proposed the relationships between *t* and *M*_*w*_ and *R* as ln*t* = 0.19+0.15*M*_*w*_+0.35ln*R* and *t* = 2.33*M*_*w*_+0.149*R*, respectively. The positive regression coefficients of *R* in the two functions illustrate the situation. However, from a physical point of view, the larger the *R* is, the smaller *t* should be. Therefore, the regression coefficient violates a law of physics but is statistically correct. In addition, ignoring the influences of site conditions on *t* as mentioned above may cause the endogenous problem, which may lead to an abnormal regression coefficient. Similarly, the reason for the abnormal effect of *H*_*n*_ on liquefaction in the casual model is the same as that for *t*. Therefore, compared with the correlation analysis method, the causal model can reflect the real impacts of factors by considering the mediation effects.

When determining the relationship between factors using the MIC method, the threshold of 0.9 times maxMIC in this study results in the omission of causal relationships between variables. For example, in the initial structure, *H*_*n*_ and *D*_*s*_ are not connected, but they share a causal connection in the subsequently modified structure. Therefore, the selection of the threshold affects the construction efficiency of the model (i.e., the number of revisions) but does not affect the structure of the final model. Therefore, using the MIC method to construct the structure of the path analysis diagram can quickly and objectively determine an initial path diagram, which greatly reduces the number of subsequent revisions, and using its structure directly as the structure of the BN also results in a strong performance.

## Conclusion

The casual path analysis method is applied for the first time to study the direct and mediation effects of various factors on earthquake-induced liquefaction in this study, and a useful approach to quantitatively identify the key factors of liquefaction is presented. The important findings are as follows:

1. Twelve key factors, *M*_*w*_, *D*_*50*_, *V*_*s*_, *PGA*, *R*, *t*, *D*_*w*_, *D*_*n*_, *D*_*s*_, *T*_*s*_, *H*_*n*_, and *FC*, are identified in this study. In addition, *I*, *V*_*s1*_, *σ*_*V*_ and $\sigma_{V}^{'}$ are multicollinearity with *PGA*, *V*_*s*_, and *D*_*s*_, respectively, but they are also important factors. The results can provide a reference for the selection of factors when constructing a predictive model for liquefaction.
2. The findings demonstrate that earthquake-induced liquefaction is a result of the comprehensive control of many factors. When considering the influences of these factors on liquefaction, focusing only on their direct effects leads to large deviations in the importance of their contributions. The 12 identified key factors, except for *t* and *V*_*s*_, possess multiple mediation paths for influencing liquefaction; of these factors, *T*_*s*_ and *D*_*n*_ are two indirect-only mediators, and *FC* and *H*_*n*_ produce suppressive effects on liquefaction. Clarifying these findings can reduce sensitivity deviations of some factors in the analysis of significant contributions and help researchers to understand the mechanism of liquefaction more clearly.
3. This paper presents a simple and effective approach for constructing a causal path structure combining MIC and correlation analysis methods and domain knowledge. The approach can greatly reduce the complexity of the model and the sample size requirement, and it can also omit the process of forming and testing a hypothesis in the construction of the causal path model. In addition, the interpretation of the causal path model can be directly used for BN model learning for liquefaction prediction. Moreover, the causal path model can also directly extract an LR model without considering the interactions between variables. The performances of these two models proved to be good upon testing with 5-fold cross-validation; however, the prediction performance of the LR model is not as good as that of the BN model.

## Supporting information

S1 Graphical abstract (TIF)

S1 File Data collected from the literature. (XLSX)
