# Supplementary figures and images for: Key factors influencing earthquake-induced liquefaction and their direct and mediation effects
Source: PLoS One. 2021 Feb 17;16(2):e0246387. doi: 10.1371/journal.pone.0246387 (PMC7888622; doi:10.1371/journal.pone.0246387)

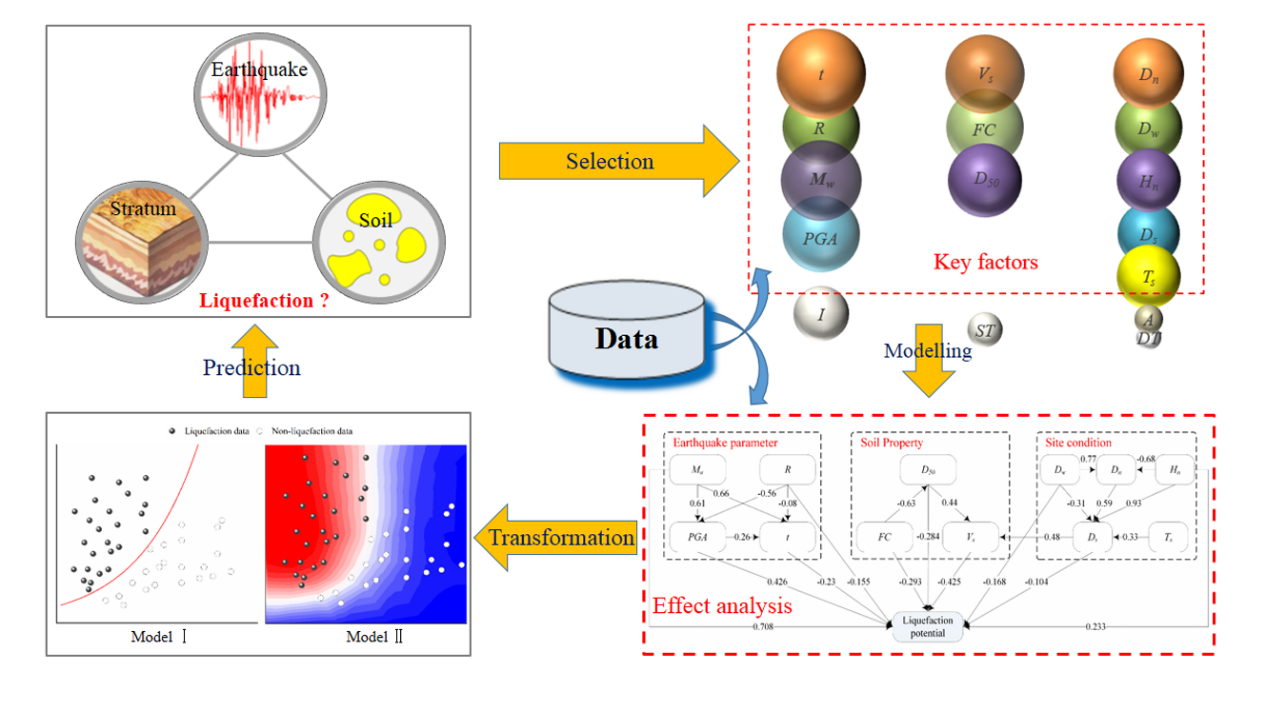

Supplement: S1 Graphical abstract — (TIF) [file pone.0246387.s001.tif]
